# Temporal shifts in semen parameters across a major COVID-19 outbreak wave: a retrospective cohort study using epidemiological time-windows

**DOI:** 10.3389/frph.2025.1691216

**Published:** 2025-09-29

**Authors:** Hai Wang, Xianjue Zheng, Haojie Pan, Jiayong Zheng, Zitong Xu

**Affiliations:** Center for Reproductive Medicine, The Third Clinical Institute Affiliated to Wenzhou Medical University, Wenzhou People’s Hospital, Wenzhou Maternal and Child Health Care Hospital, Wenzhou, China

**Keywords:** COVID-19, semen quality, conventional semen parameters, sperm acrosin activity, DNA fragmentation index

## Abstract

**Objective:**

To investigate the temporal variations in male semen quality parameters before, during, and after a major regional COVID-19 outbreak wave.

**Methods:**

This retrospective cohort study analyzed initial semen samples collected at our hospital between June 1, 2022, and June 30, 2023. Based on regional epidemiological data corresponding to a major outbreak, participants were stratified into three groups by sample collection date: a pre-outbreak group (Group A, September 1, 2022–December 31, 2022, *n* = 330), a peak-outbreak group (Group B, January 1, 2023–March 31, 2023, *n* = 413), and a post-outbreak group (Group C, April 1, 2023–June 30, 2023, *n* = 480). Conventional semen parameters, sperm acrosin activity, and sperm DNA fragmentation index (DFI) were compared.

**Results:**

The peak-outbreak group (Group B) showed significantly lower sperm concentration compared to both the pre-outbreak group (49.1 vs. 59.6 × 10^6^/ml, *P* = 0.009) and the post-outbreak group (49.1 vs. 62.6 × 10^6^/ml, *P* < 0.001). Similarly, progressive motility was significantly lower in the peak-outbreak group (37.7%) compared to the pre-outbreak (45.1%, *P* < 0.001) and post-outbreak groups (43.4%, *P* < 0.001). No significant differences were found in these parameters between the pre-outbreak and post-outbreak groups (*P* > 0.05). Sperm acrosin activity and DFI remained stable across all three groups (*P* > 0.05).

**Conclusion:**

Semen concentration and motility were significantly lower in samples collected during the peak of a major COVID-19 outbreak wave, with parameters returning to pre-outbreak levels in the subsequent 3-month period. Key sperm functional biomarkers, including acrosin activity and DNA integrity, appeared resilient throughout these distinct epidemiological phases.

## Background

In December 2019, China reported a cluster of pneumonia cases with unknown etiology to the World Health Organization. A novel β-coronavirus, named severe acute respiratory syndrome coronavirus 2 (SARS-CoV-2), which causes coronavirus disease 2019 (COVID-19), was detected in samples collected from the lower respiratory tracts of patients. This virus rapidly escalated into a global pandemic. SARS-CoV-2 infects host cells via transmembrane serine protease 2 (TMPRSS2) and the receptor angiotensin-converting enzyme 2 (ACE2) (1). The ACE2 receptor is expressed in various tissues, including the cardiovascular system, gastrointestinal tract, liver, and lungs. Damage to these organs following infection has been observed (2–4).

The human male reproductive system is susceptible to viral infections due to the incomplete barrier function of the blood-testis barrier. Multiple viruses capable of causing orchitis and male infertility (e.g., mumps virus, Zika virus, human immunodeficiency virus) have been detected in human semen. However, the impact of COVID-19 on male reproductive health remains incompletely understood (5). Studies have shown high expression levels of ACE2 in spermatogenic cells, Leydig cells, and Sertoli cells (6), suggesting that the testes may be a potential target for direct damage by SARS-CoV-2. Consequently, male reproductive health has garnered significant attention during the COVID-19 pandemic. Nevertheless, existing studies on the effects of COVID-19 on the male reproductive system are limited by small sample sizes and methodological flaws.

On December 7, 2022, the National Health Commission of China issued the “New Ten Measures” policy, which significantly relaxed COVID-19 control restrictions. This policy change led to widespread infections across cities. According to data from the Chinese Center for Disease Control and Prevention (CDC) and local hospital surveillance, the peak of the COVID-19 outbreak in Wenzhou occurred in late December 2022, marking the first large-scale transmission wave in the region where most of the population was likely infected for the first time. This public health event provided a unique ecological setting to assess population-level changes in health metrics. Against this backdrop, our study aimed to retrospectively analyze the dynamics of male semen parameters by comparing samples collected before, during, and after this major outbreak peak. We hypothesized that conventional parameters would decline during the peak transmission period and subsequently recover, while functional biomarkers like acrosin activity and the DNA fragmentation index (DFI) would remain stable.

## Methods

### Ethical approval

This study was conducted in accordance with the Declaration of Helsinki and received ethical approval from the Ethics Review Committee of Wenzhou People's Hospital (Approval No. KY-202503-056). All data were fully anonymized prior to analysis, and the requirement for individual informed consent was waived due to the retrospective nature of the study.

### Patient and public involvement

Patients or the public were not involved in the design, conduct, reporting, or dissemination plans of this research.

### Study design and setting

This was a retrospective cohort study utilizing laboratory data collected at the Center for Reproductive Medicine, Wenzhou People's Hospital, a tertiary care institution in Wenzhou, China. The center primarily serves male patients undergoing fertility evaluation. Data were retrospectively accessed from the laboratory information management system on December 20, 2023, for research purposes.

## Participants and grouping

### Eligibility criteria

We included records from all male patients who underwent their first-ever semen analysis at our center between June 1, 2022, and June 30, 2023. To minimize confounding effects, we applied the following exclusion criteria: (1) individuals with any previous semen analysis records at our center, to avoid carryover effects from potential treatments or lifestyle changes; (2) individuals with records indicating known urogenital diseases (e.g., varicocele, orchitis) or use of medications known to impact spermatogenesis; and (3) records with incomplete data for the key outcome variables.

### Grouping rationale and definitions

**Figure 1:**
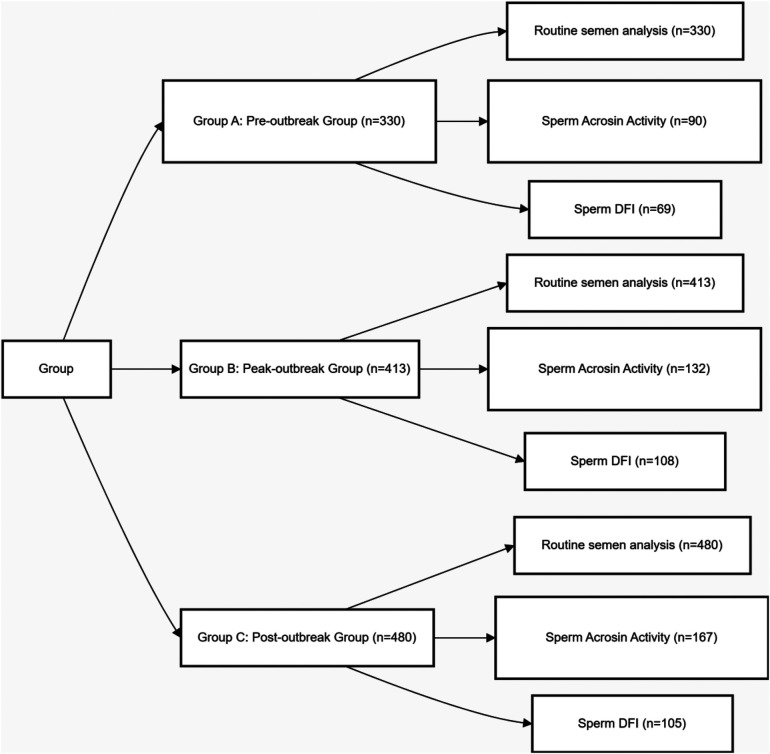
Experimental design and grouping. Due to varying clinical needs among patients, the sample sizes for sperm acrosin activity and sperm DFI measurements differ from those of the conventional semen parameters.

Participant grouping was not based on individual infection status but on the sample collection date, which was aligned with the distinct epidemiological phases of the first major SARS-CoV-2 outbreak wave in Wenzhou. This wave followed the nationwide relaxation of public health policies on December 7, 2022. Based on municipal surveillance data from the Wenzhou CDC and our hospital's SARS-CoV-2 nucleic acid testing positivity rates, which identified an infection peak in late December 2022 and January 2023, participants were stratified into three distinct temporal groups (Figure 1): Pre-outbreak Group (Group A): 330 men whose samples were collected between September 1, 2022, and December 31, 2022, representing the baseline period before the widespread community transmission. Peak-outbreak Group (Group B): 413 men whose samples were collected between January 1, 2023, and March 31, 2023, corresponding to the period of peak transmission and population-level exposure. Post-outbreak Group (Group C): 480 men whose samples were collected between April 1, 2023, and June 30, 2023, representing a post-recovery phase approximately one spermatogenic cycle after the peak outbreak.

### Data collection and variables

Data for all included participants were extracted from the laboratory information management system. All data were anonymized prior to analysis, and researchers did not have access to personally identifiable information (PII) during or after data collection. Outcome Variables: The primary outcome variables were conventional semen parameters, including semen volume (ml), sperm concentration (10^6^/ml), progressive motility (PR, %), non-progressive motility (NP, %), and immotile sperm percentage (IM, %). Secondary outcome variables were sperm functional biomarkers, including sperm acrosin activity (uIU/10^6^ sperm) and the sperm DNA fragmentation index (DFI, %). Covariate: Patient age (years) at the time of sample collection was extracted as a potential confounding variable for inclusion in statistical models.

### Laboratory procedures and assays

#### Semen sample collection and processing

Subjects were required to abstain from sexual activity for 3–7 days prior to sample collection. Whole semen samples were collected via masturbation into sterile specimen cups. Samples were immediately submitted for testing and incubated in a 37°C thermostat for ≥15 min to ensure complete liquefaction before subsequent analyses.

#### Routine semen analysis

Analyses were performed strictly according to the WHO Laboratory Manual for the Examination and Processing of Human Semen (5th edition). A Computer-Assisted Semen Analysis (CASA) system was used to quantify key parameters, including semen volume, sperm concentration, progressive motility (PR), non-progressive motility (NP), and immotile sperm percentage (IM). All tests completed internal quality control procedures.

#### Sperm acrosin activity assay

Acrosin activity was measured using a modified Kennedy method. Briefly, volumes of semen containing 7.5 × 10^6^ sperm were processed according to reagent instructions, and absorbance values were measured at 405 nm using a microplate reader to determine activity.

#### Sperm DNA fragmentation index (DFI) detection

DFI was assessed via the Sperm Chromatin Structure Assay (SCSA). Sperm samples were treated with acid and stained with acridine orange. A flow cytometer analyzed 5,000 sperm events per sample, with DFI calculated from the ratio of red (abnormal, single-stranded DNA) to green (normal, double-stranded DNA) fluorescence signals.

### Statistical analysis

Data were analyzed using Python 3.13.1. The normality of data distribution for all continuous variables was assessed using the Shapiro–Wilk test. As all key outcome variables were found to be non-normally distributed, they are presented as median and interquartile range [M (P25, P75)].

Initial comparisons of semen parameters across the three study groups (Pre-outbreak, Peak-outbreak, Post-outbreak) were performed using the Kruskal–Wallis H test to assess for an overall significant difference. If the Kruskal–Wallis test was significant, *post-hoc* pairwise comparisons were then conducted using the Mann–Whitney *U* test. A Bonferroni correction was applied to account for multiple comparisons, with a corrected *p*-value of *P* < 0.0167 (0.05/3) considered statistically significant.

To address potential confounding by demographic variables, multivariable linear regression analysis was also performed. This analysis assessed the independent association between the study group (as the primary predictor) and key semen parameters (e.g., sperm concentration, progressive motility), after adjusting for patient age.

## Results

### Comparison of routine semen parameters among groups

**Figure 2:**
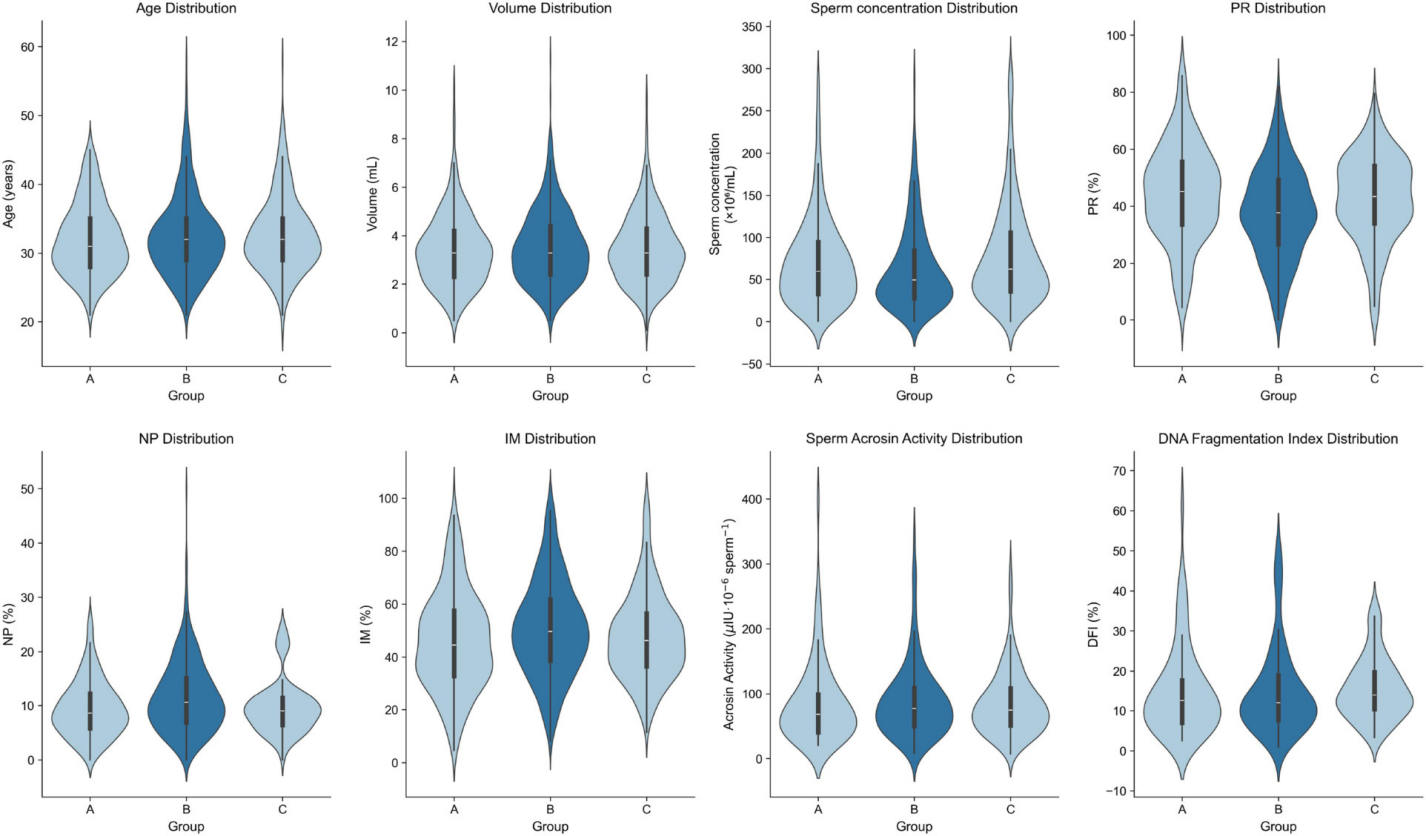
Violin plots showing data distribution of sperm parameters among the three patient groups.

Pairwise comparisons of conventional semen parameters revealed significant differences across the groups (Table 1, Figure 2). Specifically, compared to the Peak-outbreak Group, the Pre-outbreak Group exhibited significantly higher sperm concentration and progressive motility (PR), along with significantly lower non-progressive motility (NP) and immotile sperm percentage (IM) (*P* < 0.01 for all). Similarly, the Post-outbreak Group also showed significantly higher sperm concentration and PR, and significantly lower NP and IM, when compared to the Peak-outbreak Group (*P* < 0.01 for all). No statistically significant differences were detected for any of these parameters when comparing the Pre-outbreak Group and the Post-outbreak Group (*P* > 0.05), suggesting a return to baseline levels in the period following the outbreak peak.

**Table 1:** Comparison of routine semen parameters among the three groups.

Variable	Age (year)	Volume (ml)	Sperm concentration (10^6^/ml)	PR (%)	NP (%)	IM (%)
Pre-outbreak Group (A)	31.00 (28.00, 35.00)	3.30 (2.30, 4.20)	59.60 (32.55, 94.53)	45.10 (33.53, 55.55)	8.60 (5.80, 12.28)	44.55 (32.83, 57.45)
Peak-outbreak Group (B)	32.00 (29.00, 35.00)	3.30 (2.40, 4.50)	49.10 (27.70, 84.60)	37.70 (26.60, 49.20)	10.60 (6.90, 15.10)	49.60 (38.70, 61.60)
Post-outbreak Group (C)	32.00 (29.00, 35.00)	3.30 (2.40, 4.40)	62.55 (35.75, 105.85)	43.40 (33.98, 54.13)	9.00 (6.40, 11.40)	46.15 (36.45, 56.35)
Group A vs. B, *P* value	0.408	0.193	0.009*	<0.001*	<0.001*	0.001*
Group A vs. C, *P* value	0.096	0.408	0.159	0.268	0.611	0.178
Group B vs. C, *P* value	0.431	0.577	<0.001*	<0.001*	<0.001*	0.006*

* Indicates statistical significance after Bonferroni correction (*P* < 0.0167).

Data are presented as median (interquartile range). *P*-values were derived from the Mann–Whitney *U* test.

PR, progressive motility; NP, non-progressive motility; IM, immotile sperm.

### Comparison of sperm acrosin activity and DFI among groups

Pairwise comparisons of sperm acrosin activity and DNA fragmentation index (DFI) were performed across the three groups. No statistically significant differences were observed in either parameter among the groups (Table 2, Figure 2).

**Table 2:** Comparison of sperm acrosin activity and DFI among the three groups.

Variable	Sperm acrosin activity (uIU/10^6^ sperm)	Sperm DFI (%)
Pre-outbreak Group (A)	68.050 (40.4, 101.375)	12.55 (6.785, 18.31)
Peak-outbreak Group (B)	77.25 (49.75, 109.475)	11.955 (7.57, 19.0225)
Post-outbreak Group (C)	74.3 (50.6, 108.8)	13.91 (10.38, 19.715)
Group A vs. B, *P* value	0.292	0.947
Group A vs. C, *P* value	0.801	0.103
Group B vs. C, *P* value	0.396	0.058

Data are presented as median (interquartile range). *P*-values were derived from the Mann–Whitney *U* test with Bonferroni correction applied (significance threshold at *P* < 0.0167).

DFI, DNA fragmentation index.

### Multivariable regression analysis

To account for the potential confounding effect of age, a multivariable linear regression analysis was performed (Table 3). After adjusting for age, the Peak-outbreak Group (Group B) remained significantly associated with a decrease in sperm concentration (*β* = −8.492, *P* = 0.035) and progressive motility (*β* = −6.547, *P* < 0.001), and an increase in non-progressive and immotile sperm percentages compared to the Pre-outbreak Group (Group A). For the Post-outbreak Group (Group C), no significant differences in sperm concentration or progressive motility were observed when compared to the Pre-outbreak reference group. However, a significant increase in non-progressive motility and a significant decrease in immotile sperm percentage were noted. The analysis also identified age as a significant independent predictor, with increasing age being associated with a significant decrease in progressive motility and an increase in immotile sperm (*P* < 0.001 for both).

**Table 3:** Multivariable linear regression analysis for key semen parameters.

Outcome variable	Predictor	*β* (Coefficient)	95% Confidence interval	*P*-value
Sperm concentration (10^6^/ml)	Group B (vs. Group A)	−8.492	−16.401 to −0.584	0.035
Sperm concentration (10^6^/ml)	Group C (vs. Group A)	7.577	−0.087 to 15.240	0.053
Sperm concentration (10^6^/ml)	Age (year)	0.101	−0.454 to 0.657	0.72
PR (%)	Group B (vs. Group A)	−6.547	−8.863 to −4.230	<0.001
PR (%)	Group C (vs. Group A)	−1.573	−3.818 to 0.673	0.17
PR (%)	Age(year)	−0.393	−0.556 to −0.230	<0.001
NP (%)	Group B (vs. Group A)	2.11	1.143 to 3.077	<0.001
NP (%)	Group C (vs. Group A)	4.939	4.002 to 5.876	<0.001
NP (%)	Age(year)	−0.044	−0.112 to 0.024	0.202
IM (%)	Group B (vs. Group A)	4.437	1.844 to 7.030	<0.001
IM (%)	Group C (vs. Group A)	−3.366	−5.879 to −0.854	0.009
IM (%)	Age(year)	0.437	0.255 to 0.619	<0.001

β represents the change in the outcome variable for each unit change in the predictor, adjusted for other variables in the model. The Pre-outbreak Group (Group A) served as the reference category for the study groups.

## Discussion

This study revealed that key semen parameters, specifically sperm concentration and motility, were significantly lower in samples collected during the peak-outbreak period compared to those from the pre-outbreak (baseline) period. Furthermore, these parameters returned to baseline levels in the post-outbreak group, with samples collected approximately 3 months later. However, no significant changes were observed in acrosin activity or DFI. Our primary findings of a transient decline in semen quality during the peak outbreak period were robust even after controlling for age, a known confounding factor. The multivariable regression analysis confirmed that the associations between the peak-outbreak timeframe and lower sperm concentration and motility were statistically significant, independent of the patient's age. This strengthens the evidence that the observed changes are linked to the temporal events of the pandemic wave itself. Interestingly, the recovery phase demonstrated a more complex pattern than a simple return to baseline. While sperm concentration and progressive motility recovered, the persistent alterations in non-progressive and immotile sperm percentages suggest that the full restoration of spermatogenic function might involve nuanced dynamics or require a longer observation period. This finding opens new avenues for investigating the subtler, long-term impacts of widespread public health stressors on male reproductive function. Furthermore, our model confirmed the well-established negative correlation between age and sperm motility, validating the soundness of our analytical approach and its ability to dissect complex relationships within the data.

The impact of COVID-19 on the male reproductive system likely involves the following mechanisms: (1) Viral Infection and Inflammatory Response: SARS-CoV-2 may enter host cells via the ACE2 receptor pathway. Cells in the male reproductive system express ACE2 and TMPRSS2 on their surfaces, rendering these organs susceptible to SARS-CoV-2 infection (7). Direct viral damage triggers secondary inflammatory responses, such as orchitis or testicular discomfort, due to increased viral load and immune activation (8). This hypothesis is reinforced by emerging evidence of local immune system engagement within the male reproductive tract. For instance, recent work by Giugliano S. et al. has provided evidence of local immune activation and viral presence in semen, strengthening the case for a direct viral impact on testicular function (9). (2) Oxidative Stress (OS): SARS-CoV-2 Infection may impair male reproductive health by inducing systemic inflammation and oxidative stress (10). Oxidative stress, driven by excessive reactive oxygen species (ROS) production, likely plays a central role in SARS-CoV-2 mediated reproductive dysfunction, suggesting potential therapeutic value in antioxidant interventions (11). The “cytokine storm” associated with oxidative stress requires suppression of systemic inflammation for resolution (12). (3) Fever-Induced Disruption:Infection-related fever may interfere with normal reproductive physiology (13). (4) Long-Term Effects: While short-term impacts, such as erectile dysfunction and altered semen parameters, may improve over time, COVID-19 could exert prolonged effects on male reproductive function. These include potential damage to testicular spermatogenesis and disruption of hypothalamic-pituitary-gonadal (HPG) axis function (14, 15).

A prospective longitudinal cohort study found that, compared to healthy controls, COVID-19 patients exhibited significantly higher plasma levels of ACE2 enzyme, IL-1β, IL-6, IL-8, IL-10, TGF-β, TNF-α, IFN-α, IFN-γ, reactive oxygen species (ROS), caspase-8, caspase-9, and caspase-3 activity, alongside reduced superoxide dismutase (SOD) activity at baseline and during follow-up. These perturbations tended to persist over time and were associated with significant impairments in semen volume, progressive motility, sperm morphology, sperm concentration, and total sperm count (16). Another study highlighted that COVID-19 may induce histopathological or functional changes in the testes and male reproductive tract due to high ACE2 expression in these tissues, thereby mediating adverse effects on the male reproductive system (17). Previous research has similarly reported reduced semen volume, decreased total sperm count, and impaired sperm motility in patients recovering from COVID-19 (18–21). Interestingly, all observed alterations returned to baseline levels after 1–2 spermatogenic cycles post-recovery (22–25), indicating that SARS-CoV-2 induced semen parameter changes are reversible—a finding consistent with the conclusions of the current study.

However, it is important to note that the literature on this topic is not entirely uniform. Some studies have reported no significant alterations in semen parameters during the pandemic. For instance, a study by Sarier et al. (26) which compared spermiograms of infertile men before and during the COVID-19 pandemic did not find a statistically significant impact on sperm concentration or motility, suggesting that the effect may not be universal across all populations or may be influenced by other underlying factors such as infertility status. These discrepancies in findings across studies could be attributed to a variety of factors, including differences in the severity of infection, the prevalence of symptoms like fever, variations in viral strains, or distinct demographic characteristics of the study populations. Our study, which captured a population-wide acute outbreak wave, contributes to the body of evidence suggesting a transient impact, while acknowledging the heterogeneity in published reports.

Some studies have reported significant impacts of COVID-19 infection on sperm DFI, with values exceeding 30% in infected groups (27, 28). Although partial recovery occurred after 3 months of convalescence, DFI levels remained higher than those in normal control groups (29). Conversely, other researchers observed notable changes in male samples after 5 months of recovery: increased round cell counts, reduced nitrotyrosine levels, decreased total antioxidant capacity and zinc concentrations, and elevated 8-hydroxy-2′-deoxyguanosine (8-OHdG) levels in sperm. These alterations suggest that increased sperm DNA fragmentation and reduced semen quality post-COVID-19 may result from imbalances in semen pro-oxidant and antioxidant components (30). However, COVID-19 is not consistently associated with elevated DFI, implying these factors may be independent (23, 31). Some studies have also found no significant effect of COVID-19 on sperm DFI (32, 33), aligning with our findings. These discrepancies may stem from differences in viral strains or infection severity among study populations.

Sperm acrosin is a trypsin-like serine protease bound to the sperm acrosomal membrane. Acrosin activity serves as a crucial indicator of sperm's ability to penetrate the zona pellucida and represents a valuable parameter for evaluating male fertility (34–36). However, no previous studies have been identified regarding the association between SARS-CoV-2 infection and sperm acrosin activity. Our findings demonstrate that acrosin activity remains unaffected by SARS-CoV-2 infection status. This persistence may be attributed to the fact that acrosin activity primarily reflects the functional capacity of mature sperm during fertilization (35), whereas the observed decline in sperm parameters during the acute phase likely originates from spermatogenic cell damage rather than functional abnormalities in mature spermatozoa. Additionally, the limited sensitivity of the modified Kennedy method for acrosin detection might contribute to this observation. The specific mechanisms warrant further investigation.

Despite its valuable insights into population-level semen quality shifts, this study has several important limitations that must be considered when interpreting the results. First and foremost, the primary limitation is the lack of individual-level laboratory confirmation for SARS-CoV-2 infection for participants in each group. Our group stratification relied on population-level epidemiological timelines rather than individual diagnoses, a necessity driven by post-pandemic public health policy changes that discontinued mass testing. This approach introduces a significant risk of misclassification bias. For instance, some individuals in the peak-outbreak group may not have been infected, while asymptomatic infections could have occurred in the pre- and post-outbreak groups. This potential misclassification could dilute the observed associations, and it underscores that our findings reflect ecological trends rather than confirmed individual-level effects of the virus. Second, the study is susceptible to confounding from unmeasured variables. The time-window-based design introduces a risk of temporal confounding, where observed differences could be partly attributable to factors other than the COVID-19 outbreak, such as seasonal variations in semen quality, which have been previously reported. Furthermore, while we adjusted for age in our analysis, we could not control for other potential confounders such as the severity of symptoms (e.g., fever duration and intensity), lifestyle changes during the lockdown period, body mass index (BMI), or socioeconomic status, all of which could independently affect sperm quality. Third, our semen analysis was conducted according to the WHO 5th edition laboratory manual. While the 6th edition is now available and provides updated reference values and criteria, the 5th edition was the standardized protocol implemented in our laboratory throughout the entire study period. Adherence to this single standard ensured methodological consistency and comparability of data across the three time-windows, which was critical for this temporal analysis. Finally, a key limitation of this retrospective design is that routine clinical semen samples were not archived following analysis. This precluded further in-depth mechanistic investigations, such as the analysis of cytokines, viral RNA, or immune cells in semen, which could have offered valuable insights into the underlying pathophysiology. Therefore, future prospective studies should ideally be designed to include the cryopreservation of samples, thereby establishing a biobank crucial for elucidating the mechanisms of viral impacts on male reproductive health.

## Conclusion

This retrospective cohort study established time-window grouping criteria based on regional epidemiological characteristics, with the Peak-outbreak Group (Group B) being defined by the sample collection window corresponding to the population-level exposure peak identified by Wenzhou CDC and hospital-based nucleic acid testing positivity rates. corresponding to the population-level exposure peak between late December 2022 and January 2023. While the grouping strategy has inherent limitations (notably the absence of laboratory-confirmed individual infection status due to the termination of nucleic acid testing post-pandemic peak), the findings provide critical insights: Against the backdrop of widespread community transmission, acute SARS-CoV-2 infection induces significant reductions in sperm concentration and motility parameters. However, these alterations demonstrate full recovery after completion of a spermatogenic cycle (3 months), aligning with existing literature. Crucially, this study pioneers the systematic demonstration that sperm DNA fragmentation index and acrosin activity remain unaffected by SARS-CoV-2 infection status, establishing essential baseline data and addressing a critical knowledge gap in mechanistic investigations of viral impacts on male reproductive function.

## Data Availability

The datasets presented in this study can be found in online repositories. The names of the repository/repositories and accession number(s) can be found below: The data that support the findings of this study are openly available in figshare at DOI:10.6084/m9.figshare.28622210.v1.
